# Gastrointestinal stromal tumor coexisting with disseminated peritoneal leiomyomatosis

**DOI:** 10.1186/s40792-019-0690-x

**Published:** 2019-08-13

**Authors:** Kenri Akamine, Jun Kadono, Hirofumi Otsuka, Kazuto Ueno, Takeshi Shimizu, Yuki Nagata, Teruhiko Watanabe, Masahiko Osako, Naoki Ishizaki, Mineo Tabata

**Affiliations:** 1 Department of Surgery, Kagoshima Medical Association Hospital, Kagoshima, Japan; 2 Department of Obstetrics and Gynecology, Kagoshima Medical Association Hospital, Kagoshima, Japan; 3 Department of Radiology, Kagoshima Medical Association Hospital, Kagoshima, Japan; 4 Department of Pathology, Kagoshima Medical Association Hospital, Kagoshima, Japan

**Keywords:** Disseminated peritoneal leiomyomatosis, Gastrointestinal stromal tumor, Diagnosis

## Abstract

**Background:**

A case of gastrointestinal stromal tumor (GIST) coexisting with disseminated peritoneal leiomyomatosis (DPL) is rare. We report a case of GIST coexisting with DPL.

**Case presentation:**

A 50-year-old woman underwent exploratory laparoscopy under a preoperative diagnosis of gastric GIST with an ovarian tumor or peritoneal dissemination in the pelvic space. Laparoscopy showed multiple peritoneal masses in the pelvic space. Intraoperative frozen sectioning of the pelvic tumors showed multiple spindle cells, suggesting leiomyomas or retroperitoneal tumors; however, it was difficult to rule out peritoneal dissemination from GIST. No disseminated lesion was noted near GIST, and hence, we believed that GIST and pelvic lesions had different origins. We achieved R0 resection by partial resection of the stomach, total hysterectomy, and bilateral salpingo-oophorectomy. The postoperative immunohistopathological examination confirmed the final diagnosis of GIST and DPL. The patient has been recurrence free for 10 years.

**Conclusions:**

Immunohistochemical examination is essential for correct diagnosis for GIST and DPL. R0 curative resection should be scheduled after immunohistochemical examination of specimens obtained from exploratory laparoscopy.

## Background

Disseminated peritoneal leiomyomatosis (DPL) was first described in 1952 by Willson and Peale [1], and the entity was named by Taubert et al. in 1965 [2]. This rare condition is characterized by the presence of multiple smooth muscle, fibroblastic, and myofibroblastic nodules on the peritoneal surface of the pelvis and abdominal cavity [3]. Gastrointestinal stromal tumor (GIST) is a mesenchymal neoplasm derived from a precursor cell that gives rise to smooth muscle interstitial cells of Cajal in the gastrointestinal tract [4]. No case of GIST coexisting with DPL has been reported. Furthermore, the relationship between GIST and DPL is unclear. In this case report, we discuss the pathogenesis, diagnosis, and treatment strategy of the GIST and DPL.

## Case report

**Fig. 1:**
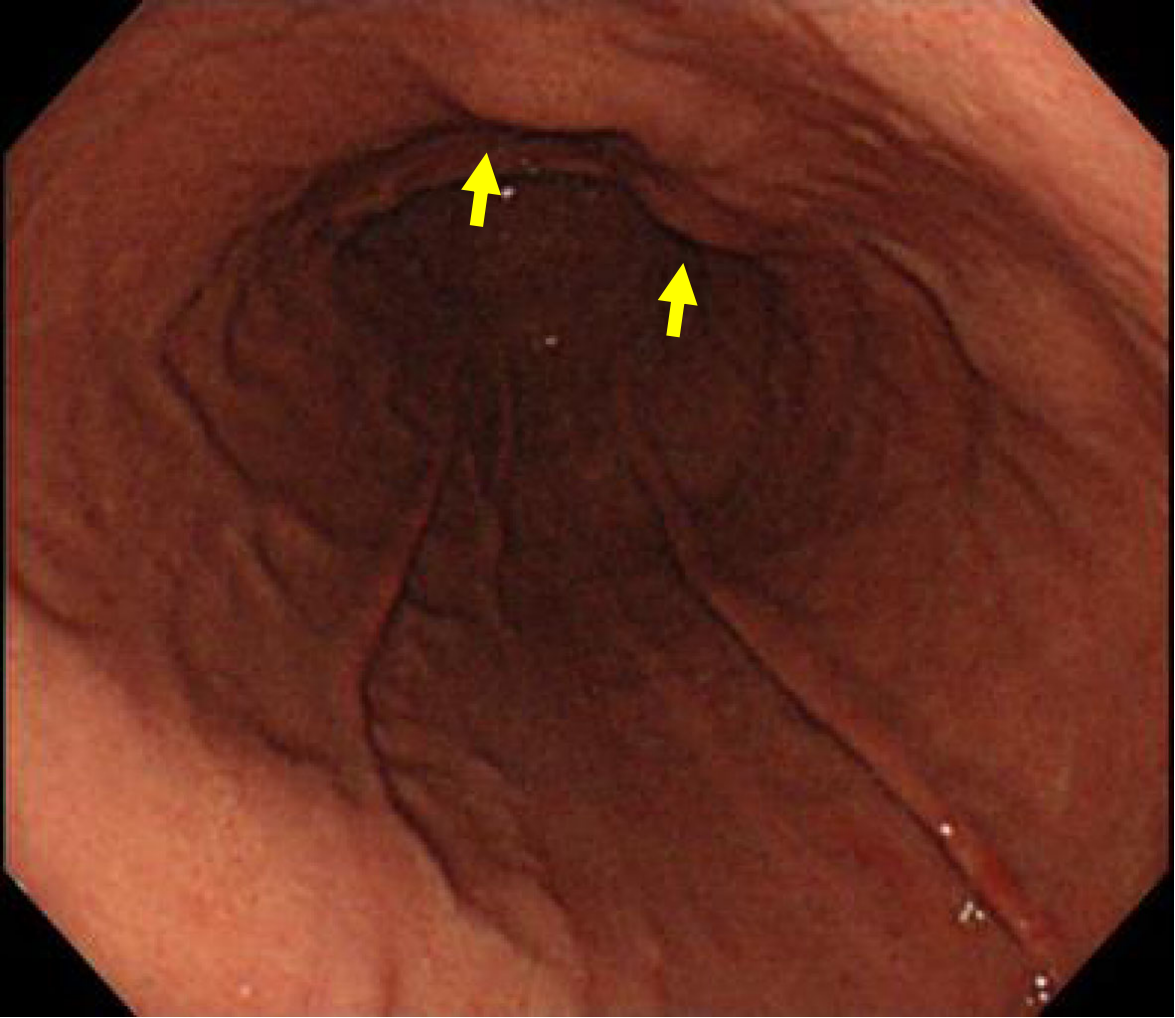
Endoscopic findings. Upper gastrointestinal endoscopy showed a submucosal tumor at the lesser curvature of the posterior body of the stomach (yellow arrow). This suggested SMT

**Fig. 2:**
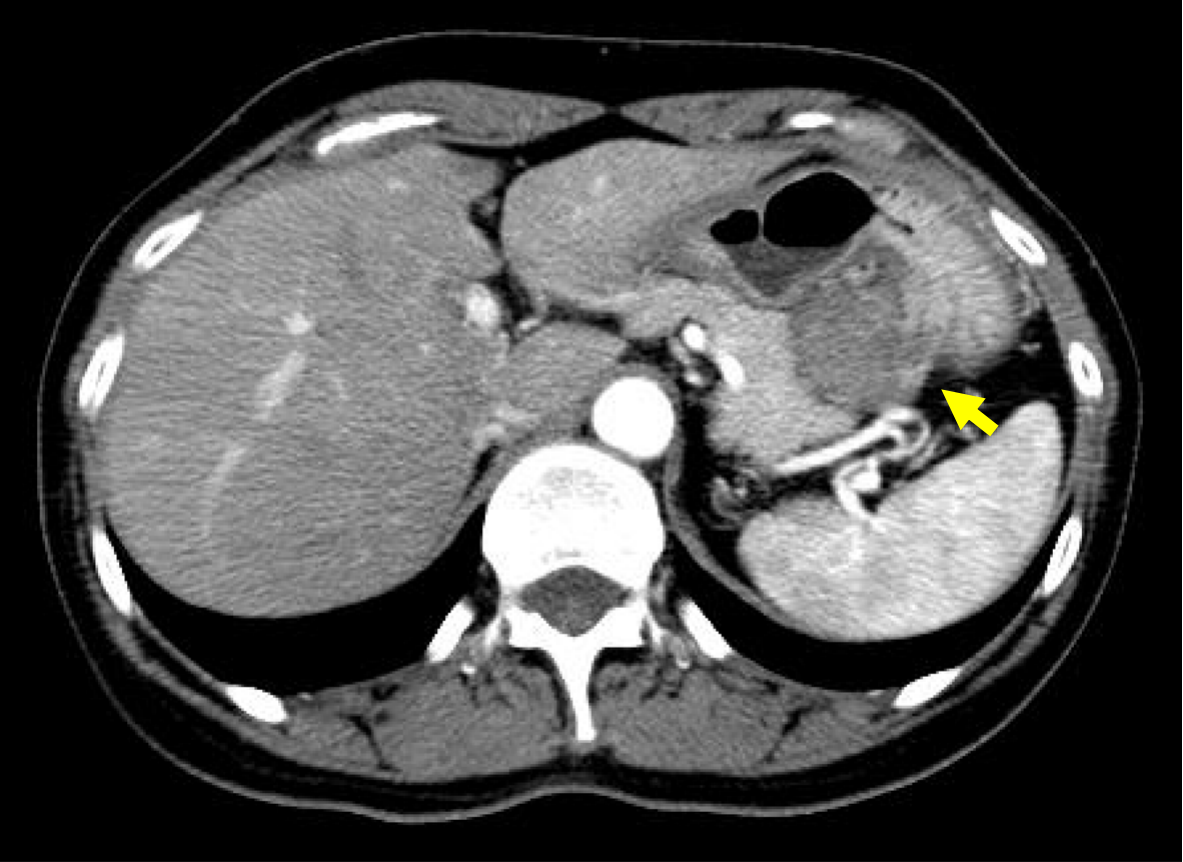
Dynamic and contrast-enhanced CT showed a 30 × 25 × 50-mm solid mass (yellow arrow) between the stomach and the pancreas tail. The lesion had a smooth margin and an inhomogeneously moderate enhancement in the arterial phase

**Fig. 3:**
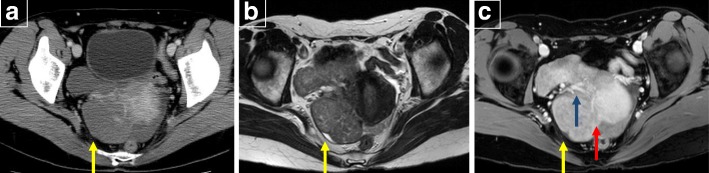
**a** CT showed the other mass (yellow arrow) in the pelvis. **b** T2-weighted MRI showed the tumor (yellow arrow) around the uterus. **c** Enhanced fat suppression T1-weighted image showing the presence of the ovarian vein (gray arrow) in this mass. The boundary between the myoma and the surrounding tissue (red arrow) was partially unclear

A 50-year-old woman presented with back pain. A gastric tumor was suspected by upper gastrointestinal series at a local clinic, and she was referred to our hospital. Upper gastrointestinal endoscopy showed a submucosal tumor in the posterior wall of the stomach body (Fig. 1). Contrast-enhanced computed tomography (CT) showed a well-demarcated inhomogeneously enhanced solid mass in the posterior wall of the stomach (Fig. 2). CT also showed a slightly enhanced lesion on the right side of the uterus (Fig. 3a). T2-weighted magnetic resonance imaging (MRI) showed a mass around the uterus (Fig. 3b). Dynamic MRI showed a gradually enhanced mass (Fig. 3c). Based on these results, a preoperative diagnosis of GIST accompanied by an ovarian tumor, leiomyoma, lymphoma, or disseminated lesion from GIST was made. Partial resection of the stomach and extended hysterectomy after exploratory laparoscopy was scheduled. The laparoscopy demonstrated a protruded tumor from the posterior wall of the stomach in the omental bursa. A soft solid tumor was observed in the broad ligament, and multiple small masses were found around the uterus. No disseminated lesion was observed near the gastric tumor in the upper abdominal space including the omental bursa. Considering these findings, we concluded that the pelvic tumors were not disseminated lesions from GIST. A tentative diagnosis of gastric GIST and ovarian tumor with disseminations was made. Intraoperative frozen sectioning of the tumor in the pelvic cavity showed spindle cells, suggesting the presence of mesenchymal tumor such as leiomyoma. However, it was difficult to rule out the presence of disseminated lesions from gastric GIST. Partial resection of the stomach was performed; thereafter, the tumors in the pelvic cavity were removed by total hysterectomy and bilateral salpingo-oophorectomy under laparotomy.

**Fig. 4:**
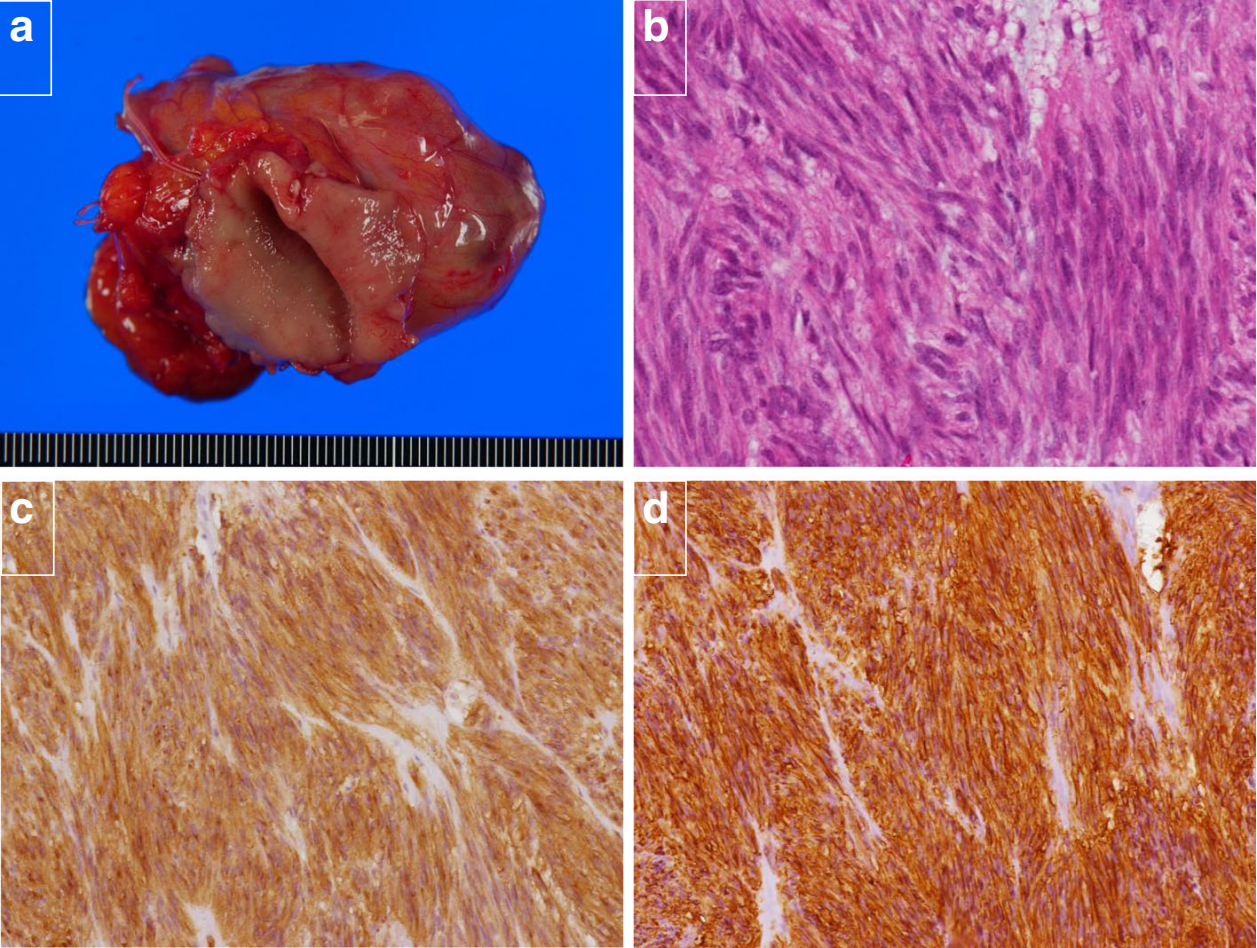
**a** Macroscopic finding of the gastric lesion. An encapsulated solid tumor measuring 45 mm in the greatest dimension. **b** Microscopic finding of the gastric lesion (hematoxylin and eosin stain [HE] × 40). The gastric tumor was composed of spindle cells forming bundles in interlacing pattern. **c** Immunohistochemical analysis of the gastric lesion. The tumor cells showed positivity for c-KIT. **d** The tumor cells showed positivity for CD34

**Fig. 5:**
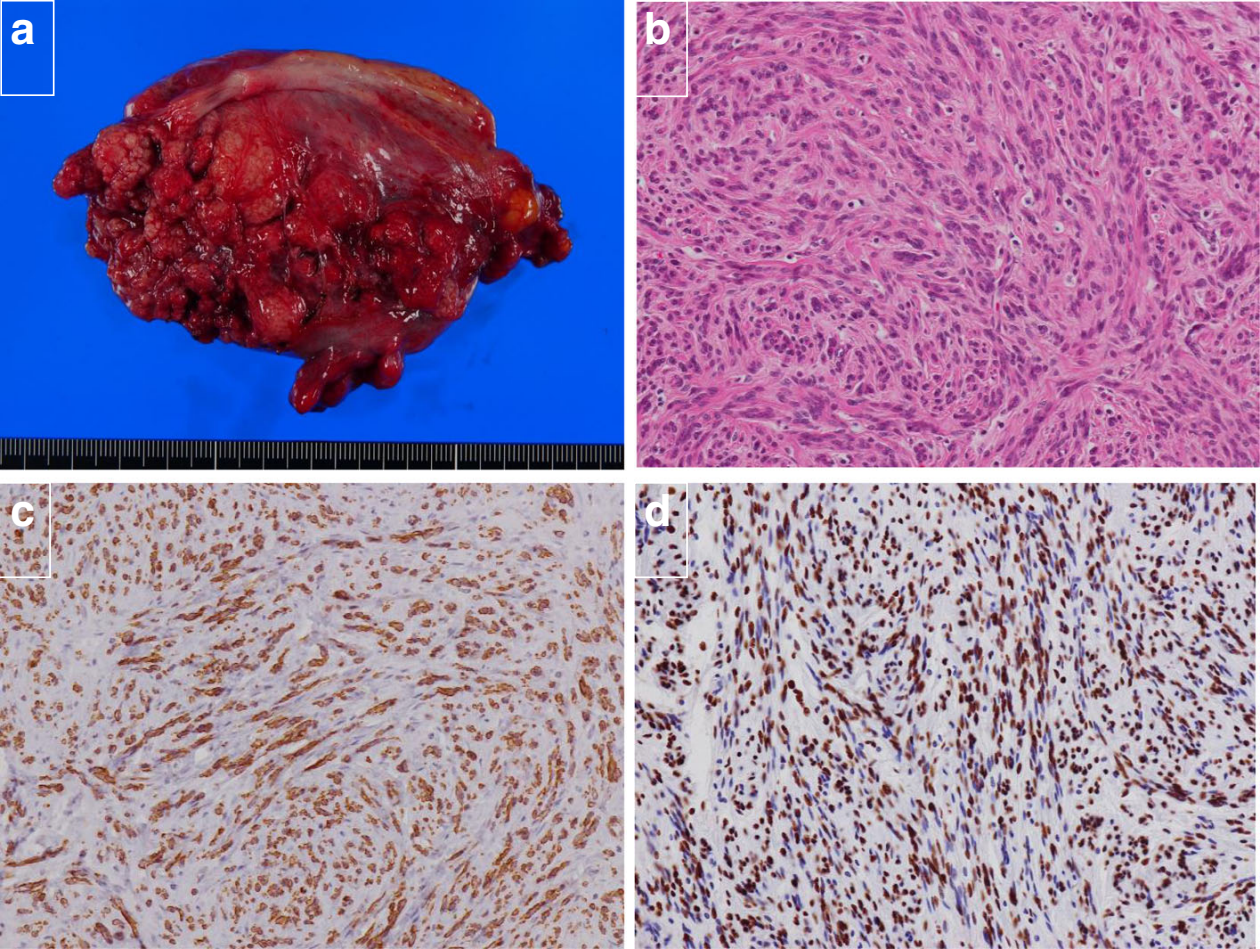
**a** Macroscopic finding of the tumor of broad ligament. A tumorous mass composed of numerous ill-defined or fused small nodules. **b** Microscopic finding of the tumor of broad ligament (HE × 20). Spindle cells with eosinophilic cytoplasm that resemble smooth muscle cells. **c** Immunohistochemical analysis of the pelvic lesion. They were positive for smooth muscle actin. **d** They were positive for ER

The resected gastric specimen showed an encapsulated solid tumor measuring 45 mm in the greatest dimension (Fig. 4a). Microscopically, the gastric tumor was composed of spindle cells forming bundles in an interlacing pattern (Fig. 4b). Upon immunohistochemical analysis, the tumor cells showed positivity for c-KIT and CD34 (Fig. 4c, d) and very low positivity for Ki-67 and negativity for actin, desmin, HHF-35, S-100 protein, estrogen receptor (ER), and progesterone receptor (PgR) (Table 1). These findings were identical for GIST. Meanwhile, the tumor of the broad ligament showed ill-defined or fused small nodules (Fig. 5a). Microscopically, the nodules of the broad ligament, uterus, and its surrounding peritoneum were composed of spindle cells resembling smooth muscle cells (Fig. 5b). Immunohistochemically, they were positive for smooth muscle actin, desmin, HHF-35, ER, and PgR (Fig. 5c, d), but negative for S-100 protein, CD34, and c-KIT (Table 1). The pelvic tumors were diagnosed as DPL based on these immunohistopathological results. The risk grade of GIST was low because the mitotic counts of GIST were < 5 per 50 HPF. The postoperative course was uneventful. The patient had no complications and was discharged on the 14th day after surgery. Subsequently, the patient was followed up at 6-month intervals for the first 5 years and then annually for another 5 years. She has neither had any recurrence nor received any drug therapy for 10 years.

**Table 1:** Immunohistochemical comparison of GIST and DPL

	c-KIT	CD34	Actin	Desmin	HHF-35	S-100 protein	ER	PgR
The gastric lesion	+	+	−	−	−	−	−	−
The pelvic lesion	−	−	+	+	+	−	+	+

+ positive, − negative, *ER* estrogen receptor, *PgR* progesterone receptor

## Discussion

Various hypotheses regarding the pathogenetic mechanism of DPL have been reported; however, it remains uncertain. It is probable that DPL is developed from smooth muscle differentiation of the submesothelial multipotential stem cells, which is a common source of origin of the secondary müllerian system [3]. However, it has been unclear whether the stimuli to induce the differentiation is hormonal, genetic, or both [5]. It was described that in 70% of cases, the underlying cause of hormonal alteration is pregnancy, and in the remaining cases, the causes are oral contraceptives or hormone-secreting tumors [6]. These results suggested that increased sensitivity to estrogen predisposes the stem cells to the development of DPL. However, occasionally reported cases in men and postmenopausal women, and the normal hormone levels indicate the involvement of other unknown factors [7–9]. In this case report, the patient was a premenopausal woman. However, we did not examine her serum estrogen level. Further investigation of such cases and genetic analysis are required in the future.

DPL is pathologically benign smooth muscle tumors in which nuclear atypia is uncommon and mitotic figures are scarcely seen [10]; however, some cases of DPL showed recurrent and malignant transformation [7–9]. Based on PubMed search from 1985 to 2018, malignant transformation has been reported in 19 cases and the prognosis for the patients is approximately 1 to 12 months. The cases with the histological images of high cell density, aggregated atypical nuclei, and frequent mitoses were diagnosed as malignancy. The mechanism of malignant transformation of DPL remains unknown [11, 12]. However, DPL with uterine leiomyomas has a low risk potential for malignant transformation, whereas DPL without exposure to estrogen, without uterine leiomyomas, and without ER and PgR expression may have a high malignant potential [7]. Additionally, the several instances of huge tumor and recurrence are an indicator of malignancy [13].

Reported treatment modalities include surgical resection and hormonal therapies [12]. Hormonal therapies such as anti-estrogen therapy are recommended when DPL is diagnosed in association with pregnancy, exogenous estrogen exposure, or uterine leiomyomas [7]. Abortion during pregnancy and discontinuing oral contraceptives are anti-estrogen therapies [12]. The effectiveness of gonadotropin-releasing hormone agonist was also reported [14]. Bilateral salpingo-oophorectomy was shown to be effective in cases without hormonal abnormalities [12, 15]. In cases with a small number of DPL tumors, R0 resection should be considered. In cases with a large number of DPL tumors that are symptomatic, a reduction surgery for symptomatic relief and postoperative hormonal therapies such as anti-estrogen therapies are recommended [16, 17]. Recurrence would probably not occur in the patient described in our case, as the tumor was resectable with ER and PgR expression. High-risk cases of malignancy require sufficient resection and close follow-up during the first year after diagnosis [7]. Some malignant cases were placed on polychemotherapy with doxorubicin, ifosfamide (IFX), etoposide, cyclophosphamide, and cisplatin. However, these therapies had no favorable results [8, 11]. Conversely, Raspagliesi et al. [18] reported a malignant case, in which the EID regimen with IFX, dacarbazine, and epirubicin had an effective result [12]. The standard chemotherapy has not been established.

GIST is a mesenchymal neoplasm derived from a precursor cell that differentiates into smooth muscle interstitial cells of Cajal in the gastrointestinal tract by c-KIT mutation or overexpression [4]. Tazawa et al. [19] examined the tumors of GIST and revealed a bi-or triphenotypic differentiation from precursor cells toward Cajal cells, smooth muscle, and/or Schwann cells. They also reported GIST of pure Cajal cell type was seen rather infrequently: nine (16%) of the 58 lesions. Besides, to the best our knowledge, GIST was not reported as estrogen-induced tumor. In our case, since the gastric tumor, which was diagnosed as GIST, was negative for ER and PgR, we believed that the tumor was not associated with estrogen. The GIST was positive for c-KIT and CD34, and negative for actin, desmin, HHF-35, ER, and PgR. However, the DPL was positive for ER, PgR, actin, desmin, and HHF-35, and negative c-KIT and CD34, which revealed no immunohistochemical relationship between the GIST and DPL. Thus, the coexistence of GIST and DPL in the patient described in this case report could be coincidental. We could diagnose our case by immunohistochemical examinations. However, if the postoperative diagnosis is difficult even by immunohistochemical examinations, KIT and platelet-derived growth factor-alpha (PDGFRA) genotyping is useful. KIT mutations in GIST occur mainly in the exon 11 followed by exon 9 and have been identified in exons 13 and 17. These mutations are associated with sensitivity to tyrosine kinase inhibitors and tumor aggressiveness [20]. In our case, the risk grade of GIST was low. However, in cases with a risk of recurrence and cases of progressive GIST, KIT and PDGFRA genotyping is important for the assessment of drug sensitivity and tumor aggressiveness [20].

For cases in which GIST and DPL coexist, diagnosis and treatment strategy were discussed. Because both GIST and DPL have various patterns in terms of form, tumor homogeneity, density, enhancement, and boundary clarity in enhanced CT and MRI, preoperative diagnosis is difficult. Similar macroscopic and histological findings in GIST and DPL also make it difficult to diagnose even by intraoperative frozen section analysis; therefore, immunohistochemical examination is essential. Recently, endoscopic ultrasonography-guided fine-needle aspiration biopsy has enabled the diagnosis of GIST. Nevertheless, the preoperative diagnosis of pelvic lesion remains difficult. Transvaginal ultrasonography-guided fine-needle aspiration biopsy has some risks of dissemination in malignant cases. Exploratory laparoscopy would be useful for diagnosing of pelvic lesion with difficult differential diagnosis [21]. In our case, it was difficult to rule out GIST from DPL even by intraoperative frozen section analysis. However, no disseminated lesion was observed near the gastric tumor in the upper abdominal space including the omental bursa. Based on these findings, we believed that the pelvic tumors were not disseminated from gastric GIST but from the ovarian or uterus primary lesions. Thereafter, the R0 resection was performed. Total hysterectomy and bilateral salpingo-oophorectomy could have been unnecessary if pelvic lesions had been diagnosed as dissemination from GIST. In our case, R0 curative resection after examination of tumor localization and immunohistochemical analysis of specimens from exploratory laparoscopy is the best therapeutic strategy.

## Conclusions

Immunohistochemical examination is essential for correct diagnosis for GIST and DPL. In the present case, GIST and DPL seemed to be derived from different origins based on the immunohistochemical examination. R0 curative resection should be scheduled after immunohistochemical examination of specimens obtained from exploratory laparoscopy.

## Data Availability

All datasets supporting the conclusions of this article are included within the article.

## Abbreviations

CT: Computed tomography
DPL: Disseminated peritoneal leiomyomatosis
ER: Estrogen receptor
GIST: Gastrointestinal stromal tumor
HE: Hematoxylin and eosin stain
MRI: Magnetic resonance imaging
PDGFRA: Platelet-derived growth factor-alpha
PgR: Progesterone receptor
